# Supplementary material for: Adaptation and Psychometric Properties of the Behavioral Regulation in Exercise Questionnaire (BREQ-3) for Motivation Towards Incidental Physical Activity
Source: Behav Sci (Basel). 2025 Jan 23;15(2):114. doi: 10.3390/bs15020114 (PMC11852020; doi:10.3390/bs15020114)
Supplement: Supplementary file 1 [file behavsci-15-00114-s001.zip › Table S1.pdf]

Adaptación del Cuestionario de Regulación Conductual en el Ejercicio (BREQ-3) para la motivación hacia la actividad física incidental.

Para responder el siguiente cuestionario, considere únicamente aquellas actividades físicas realizadas en la vida diaria, es decir, las realizadas en el hogar, en el estudio o trabajo, y en el tiempo libre, que no tienen fines de salud o fitness. Por ejemplo, activades como utilizar escaleras, caminar o andar en bicicleta, jardinería o tareas domésticas, actividades en horario de clases o de trabajo, pasear al perro o a las mascotas, ir de compras, actividades de juegos para niños, entre otras. Al responder el cuestionario no se deben tener en cuenta el ejercicio físico realizado en gimnasios, talleres deportivos, o deportes a nivel competitivo o recreativo.

INSTRUCCIONES: Seleccione una de las opciones de respuesta según qué tan ciertas sean para usted las siguientes afirmaciones.

Ítems	Enunciado	Nada verdadero	Algo verdadero	Medianamente verdadero	Mayormente verdadero	Totalmente verdadero
1	Yo hago actividad física porque los demás me dicen que debo hacerlo.					
2	Yo hago actividad física porque me siento culpable cuando no lo hago.					
3	Yo hago actividad física porque valoro los beneficios que tiene realizarla.					
4	Yo hago actividad física porque creo es divertida.					
5	Yo hago actividad física porque está de acuerdo con mi forma de vida.					
6	Yo hago actividad física, pero no veo por qué tengo que hacerla.					
7	Yo hago actividad física porque mis amigos/familia/pareja me dicen que debo hacerlo.					
8	Yo hago actividad física porque me siento avergonzado/a si no lo hago.					
9	Yo hago actividad física porque para mí es importante hacer actividad física regularmente.					
10	Yo hago actividad física porque considero que la actividad física forma parte de mí.					
11	Yo hago actividad física, pero no veo por qué tengo que molestarme en hacerla.					
12	Yo hago actividad física porque disfruto realizarla.					
13	Yo hago actividad física porque otras personas no estarán contentas conmigo si no hago actividad física.					
14	Yo hago actividad física, pero no veo el sentido que tiene hacer actividad física.					
15	Yo hago actividad física porque veo la actividad física como una parte fundamental de lo que soy.					
16	Yo hago actividad física porque siento que he fallado cuando no he realizado un rato de actividad física.					
17	Yo hago actividad física porque pienso que es importante hacer el esfuerzo de hacer actividad física regularmente.					
18	Yo hago actividad física porque encuentro que es una actividad agradable.					
19	Yo hago actividad física porque me siento bajo la presión de mis amigos/familia para realizar actividad física.					
20	Yo hago actividad física porque considero que está de acuerdo con mis valores.					
21	Yo hago actividad física porque me pongo nervioso/a si no realizo actividad física regularmente.					
22	Yo hago actividad física porque me resulta placentero y satisfactorio realizarla.					
23	Yo hago actividad física, pero pienso que realizarla es una pérdida de tiempo.					
