# Supplementary material for: Adaptation and Psychometric Properties of the Behavioral Regulation in Exercise Questionnaire (BREQ-3) for Motivation Towards Incidental Physical Activity
Source: Behav Sci (Basel). 2025 Jan 23;15(2):114. doi: 10.3390/bs15020114 (PMC11852020; doi:10.3390/bs15020114)
Supplement: Supplementary file 1 [file behavsci-15-00114-s001.zip › Table S2.pdf]

Adaptation of the Exercise Behavioural Regulation Questionnaire (BREQ-3) for motivation towards incidental physical activity.

To answer the following questionnaire, consider only those physical activities performed in daily life, i.e., those performed at home, at study or work, and in free time, that are not for health or fitness purposes. For example, activities such as using stairs, walking or cycling, gardening or housework, activities during school or work hours, walking the dog or pets, shopping, and children's play activities, among others. When answering the questionnaire, physical exercise performed in gyms, sports workshops, or sports at a competitive or recreational level should not be taken into account.

INSTRUCTIONS: Select one of the answer options based on how true the following statements are to you.

Items	Statement	Nothing true	Something true	Medium true	Mostly true	Totally true
1	I do physical activity because others tell me that I must do it.					
2	I do physical activity because I feel guilty when I do not.					
3	I do physical activity because I value the benefits of doing it.					
4	I do physical activity because I think it's fun.					
5	I do physical activity because it is in accordance with my way of life.					
6	I do physical activity, but I don't see why I have to do it.					
7	I do physical activity because my friends/family/partner tell me that I should.					
8	I do physical activity because I feel embarrassed if I do not.					
9	I do physical activity because it is important for me to do physical activity regularly.					
10	I do physical activity because I consider physical activity to be part of me.					
11	I do physical activity, but I don't see why I have to bother doing it.					
12	I do physical activity because I enjoy doing it.					
13	I do physical activity because other people will not be happy with me if I do not do physical activity.					
14	I do physical activity, but I do not see the point of doing physical activity.					
15	I do physical activity because I see physical activity as a fundamental part of who I am.					
16	I do physical activity because I feel that I have failed when I have not done a while of physical activity.					
17	I do physical activity because I think it is important to make an effort to do physical activity regularly.					
18	I do physical activity because I find it to be an enjoyable activity.					
19	I do physical activity because I feel under pressure from my friends/family to be physically active.					
20	I do physical activity because I consider it to be in accordance with my values.					
21	I do physical activity because I get nervous if I do not do physical activity regularly.					
22	I do physical activity because it is pleasant and satisfying for me to do it.					
23	I do physical activity, but I think that doing it is a waste of time.					
